# Head-to-Head Comparison of Two Popular Cortical Thickness Extraction Algorithms: A Cross-Sectional and Longitudinal Study

**DOI:** 10.1371/journal.pone.0117692

**Published:** 2015-03-17

**Authors:** Alberto Redolfi, David Manset, Frederik Barkhof, Lars-Olof Wahlund, Tristan Glatard, Jean-François Mangin, Giovanni B. Frisoni, neuGRID Consortium, for the Alzheimer’s Disease Neuroimaging Initiative

**Affiliations:** 1 Laboratory of Epidemiology & Neuroimaging, IRCCS San Giovanni di Dio Fatebenefratelli, Brescia, Italy; 2 Gnúbila France, Imp Pres d’en Bas, Argonay, France; 3 Department of Radiology, VU University Medical Center, Amsterdam, The Netherlands; 4 Department of Neurobiology, Caring Sciences & Society, Division of Clinical Geriatrics Novum, Karolinska Institutet, Stockholm, Stockholm, Sweden; 5 CREATIS, CNRS, INSERM, University of Lyon, Lyon, France; 6 McConnell Brain Imaging Centre, Montreal Neurological Institute, McGill University, Montreal, Canada; 7 CATI, Neurospin, CEA, GIF/YVETTE, France; 8 Laboratory of Neuroimaging of Aging, Memory Clinic and LANVIE, University Hospitals and University of Geneva, Geneva, Switzerland; University of Muenster, GERMANY

## Abstract

**Background and Purpose:**

The measurement of cortical shrinkage is a candidate marker of disease progression in Alzheimer’s. This study evaluated the performance of two pipelines: Civet-CLASP (v1.1.9) and Freesurfer (v5.3.0).

**Methods:**

Images from 185 ADNI1 cases (69 elderly controls (CTR), 37 stable MCI (sMCI), 27 progressive MCI (pMCI), and 52 Alzheimer (AD) patients) scanned at baseline, month 12, and month 24 were processed using the two pipelines and two interconnected e-infrastructures: neuGRID (https://neugrid4you.eu) and VIP (http://vip.creatis.insa-lyon.fr). The vertex-by-vertex cross-algorithm comparison was made possible applying the 3D gradient vector flow (GVF) and closest point search (CPS) techniques.

**Results:**

The cortical thickness measured with Freesurfer was systematically lower by one third if compared to Civet’s. Cross-sectionally, Freesurfer’s effect size was significantly different in the posterior division of the temporal fusiform cortex. Both pipelines were weakly or mildly correlated with the Mini Mental State Examination score (MMSE) and the hippocampal volumetry. Civet differed significantly from Freesurfer in large frontal, parietal, temporal and occipital regions (p<0.05). In a discriminant analysis with cortical ROIs having effect size larger than 0.8, both pipelines gave no significant differences in area under the curve (AUC). Longitudinally, effect sizes were not significantly different in any of the 28 ROIs tested. Both pipelines weakly correlated with MMSE decay, showing no significant differences. Freesurfer mildly correlated with hippocampal thinning rate and differed in the supramarginal gyrus, temporal gyrus, and in the lateral occipital cortex compared to Civet (p<0.05). In a discriminant analysis with ROIs having effect size larger than 0.6, both pipelines yielded no significant differences in the AUC.

**Conclusions:**

Civet appears slightly more sensitive to the typical AD atrophic pattern at the MCI stage, but both pipelines can accurately characterize the topography of cortical thinning at the dementia stage.

## Introduction

Structural imaging has had a long role as biomarker of progression among entry criteria for AD trials [1]. The advent of disease-modifying therapies has led to interest in the use of magnetic resonance imaging (MRI) as a possible “surrogate” measure of outcome. The two most established markers of progression on MRI are the hippocampal and the whole brain atrophy rates [2]. However, the first study assessing the effects of β-amyloid immunotherapy reported surprising findings, i.e. greater hippocampal and whole-brain atrophy rates in patients treated with AN1792 vaccination [3]. On the contrary, cortical thickness might be a promising “global” measure of disease progression, as it could represent a marker more specifically related to the evolution of AD evolution [4,5] and might be useful to evaluate the efficacy of new disease-modifying therapies [6].

Several tools for the automatic extraction of cortical thickness have been developed, each based on different levels of complexity, robustness, and automation. Among others, the Civet-CLASP pipeline [7] and Freesurfer [8] are the two most exploited algorithms within the neuroscientific community. Obtaining an accurate thickness measurement requires the explicit reconstruction of the outer boundary on the base of the inner boundary [9], which can be done along two different approaches: (I) a skeleton method or (II) a model-based deformation of the inner surface. CIVET makes use of the skeleton mesh-based approach called constrained Laplacian anatomic segmentation using proximity. The pial surface is expanded from the white surface up to the boundary between gray matter and CSF, along a Laplacian map [10]. Terms for stretch and self-proximity are included to regularize the deforming mesh and avoid mesh self-intersection inside sulci. Differently, Freesurfer makes use of iterative and adaptive deformation and segmentation methods, deforming the mesh to reconstruct the inner and the pial surfaces. Freesurfer uses a routine function to find and correct the topological defects in the initial inner surface. The deformable model is constrained by a second-order smoothing term [11] and by a mesh self-intersection prevention routine [8], which both help to correctly establish the boundaries between adjacent banks in tight sulci. Unfortunately, some relevant problems hamper the use of these techniques. Both tools measure the cortical thickness from two 3D cortical sheets, each of which is composed by thousands of vertices and faces, making the reconstruction of the cortical mantle a complex and time consuming procedure [12].

Although several methods have been proposed in the past decades, little work has been done to compare their performances on real clinical datasets [13]. The aim of this study was to perform a head-to-head comparison between Civet-CLASP and Freesurfer. This can be considered a mandatory step toward the standardization of cortical thickness biomarkers, which in turn will pave the way to effectively translate a three-dimensional cortical marker to innovative disease modifying trials.

## Materials and Methods

### Subjects

The sample group we selected consisted of 185 subjects (69 normal elderly controls (CTR), 37 stable MCI (sMCI), 27 progressive MCI (pMCI), and 52 Alzheimer (AD) patients), belonging to the Alzheimer’s Disease Neuroimaging Initiative (ADNI1). Demographics and clinical data are summarized in Table 1. MMSE and CDR scores differed significantly among the four groups (P<0.001), while age and educational levels were not significantly different. There was a significant difference in sex (P < 0.002) with a higher prevalence of male. Data used in preparation of this article were obtained from the Alzheimer’s Disease Neuroimaging Initiative (ADNI) database. As such, the investigators within the ADNI contributed to the design and implementation of ADNI and/or provided data but did not participate in analysis or writing of this report. ADNI1 study is conducted in accordance with the Good Clinical Practice guidelines, the Declaration of Helsinki, and U.S. 21 CFR Part 50 (Protection of Human Subjects), and Part 56 (Institutional Review Boards). ADNI1 study was approved by the Institutional Review Boards (IRB) of all of the participating institutions. Specifically, they are: Albany Medical College, Banner Alzheimer’s Institute, Baylor College of Medicine, Boston University, Brigham and Women’s Hospital, Butler Hospital Memory & Aging Program, Case Western Reserve University, Cleveland Clinic, Columbia University, Darthmouth—Hitchcock Medical Center, Dent Neurologic Institute, Duke University Medical Center, Emory University, Georgetown University, Howard University, Indiana University, Jefferson Hospital for Neuroscience, Johns Hopkins University, Mayo Clinic, Jacksonville, Mayo Clinic, Rochester, McGill University/Jewish General Hospital Memory Clinic, Medical University of South Carolina, Mount Sinai School of Medicine, Neurological Care of Central New York, New York University Medical Center, Northwestern University, Ohio State University, Olin Neuropsychiatry Research Center, Oregon Health and Science University, Parkwood Hospital, Premiere Research Institute, Rhode Island Hospital, Rush University Medical Center, Saint Joseph’s Health Center, Stanford University, Banner Sun Health Research Institute, Sunnybrook Health Sciences, University of Alabama, Birmingham, University of British Columbia, University of California, Davis, University of California, Irvine, University of California, Irvine-BIC, University of California—Los Angeles, University of California—San Diego, University of California—San Francisco, University of Kansas, University of Kentucky, University of Michigan, Ann Arbor, University of Nevada School of Medicine, Las Vegas, University of Pennsylvania, University of Pittsburgh, University of Rochester, University of Southern California, University of Texas Southwestern Medical Center, University of Wisconsin, Wake Forest University, Washington University St. Louis, Wein Center for Clinical Research and Yale University School of Medicine. Informed written consent was obtained from all participants at each site. A detailed description of the study procedures, IRB approval and informed written consents is available at http://www.adni-info.org/pdfs/adni_protocol_9_19_08.pdf (section D.5). Data used in this analysis were downloaded from the ADNI database (http://adni.loni.usc.edu/). List of subjects’ RIDs can be found in S1 Table.

**Table 1:** Demographic and clinical characteristics.

	CTR	sMCI	pMCI	AD	P
***Number***	69	37	27	52	
***Age (y)***	75.6 ± 4.8	74.6 ± 7.5	73.1 ± 8	76.0 ± 6.2	N.S.
***Education (y)***	15.9 ± 2.9	15.6 ± 3.3	16.6 ± 2.1	15.0 ± 2.6	N.S.
***Gender (M/F)***	38/31	22/15	17/10	27/25	0.002
***MMSE (BSL)***	29.2 ± 1.0	27.4 ± 2.0	27.1 ± 1.7	23.4 ± 2.3	<0.001
***Δ MMSE***	0.1 ± 1.4	- 0.4 ± 1.8	- 3.4± 3.6	- 3.9 ± 5.1	<0.001
***CDR***	0 (69)	0.5 (37)	0.5 (27)	0.5 (27)–1 (25)	<0.001
***ApoE ε4 carriers (%)***					
***-/-***	66.5	35	40.5	31	
***-/+***	28	60	44.5	50	
***+/+***	5.5	5	15	19	

Data are expressed as mean value ± standard deviation (σ). BSL: Baseline; Δ: Difference between month 24 and baseline; MMSE: Mini Mental State Examination scores; CDR: Clinical Dementia Ratings score; CTR: Controls; sMCI: stable MCI; pMCI: progressive MCI; AD: Alzheimer’s Disease; P: significance on Fisher’s exact test or ANOVA; N.S.: not significant.

### Research infrastructures and pipelines

The evaluation of the cortical thickness is a computationally demanding task. We used two online e-infrastructures, namely neuGRID (https://neugrid4you.eu) [14] and VIP (http://vip.creatis.insa-lyon.fr) [15] to massively distribute job analyses, thus reducing the overall processing. Civet’s and Freesurfer’s main features are summarized as follow:

- Civet-CLASP uses an iterative morphing method and intensity non-uniformity correction; spatial normalization to stereotaxic space; tissue classification; cortical surface extraction; cortical thickness measurement. The correspondence among subjects is granted by the nonlinear registration of the sulcal geodesic depth map with an average sulcal depth sphere surface [10].
- Freesurfer uses iterative adaptative morphing/segmentation methods and relies on similar preprocessing steps, although differently arranged. The white matter derives from the segmentation and topology correction. Gray matter is derived along T1 intensity gradient. Correspondence among subjects is obtained through surface registration to the Freesurfer reference atlas. In this study, we used the longitudinal processing stream, where the variability is reduced using repeated measures from the same subject (i.e.: baseline, month 12 (data not shown), and month 24 cross-sectional analyses) as common information to initialize the process [16].

Table 2 reports the main features of the two pipelines.

**Table 2:** Comparative table.

FEATURES	CIVET	FREESURFER
**MRI input file format**	MINC	DICOM; NIFTI
**Mesh output format**	MNI OBJ	PIAL
**3D mesh generation**	Iterative morphing method (i.e.: skeleton-based reconstruction). The pial surface is expanded from the white surface to the boundary between gray matter and CSF along a Laplacian map	Iterative adaptive morphing and segmentation methods (i.e.: model-based deformation of the inner surface)
**3D mesh pitfalls**	Requiring corrections for topological errors	Affected by geometric inaccuracies
**Sensitivity to artifacts**	Variation of the signal intensity across the image; shading artifacts; intensity non-uniformity (through N3 procedure); poor radio frequency field uniformity, and eddy currents are mitigated	Motion correction (when there are multiple MR source volumes of the same subject) and non-uniform intensity normalization in MR data (through N3 procedure) are carried out
**Cortex representation**	Geometrically accurate	Topologically correct
**Longitudinal stream**	Option not available	Option available. Freesurfer, during the longitudinal stream, through repeated cross-sectional measures from the same subject reduces the variability of the cortical thickness estimation. In the present study the longitudinal stream has been used (i.e.: baseline, month 12, and month 24)
**Computational time**	≅7 hours per single subject	≅35 hours per single subject
**Intra-algorithm thickness repeatability**	High	High
	(no differences between MPRAGE and MPRAGE repeat FDR corrected p-maps)	(no differences between MPRAGE and MPRAGE repeat FDR corrected p-maps)
**Number of vertices on the cortical surface**	ICO6	ICO7
	(# Vertices = 2*(10*4^n+2) = 81′924 vertices)	(# Vertices = 2*(10*4^n+2) = 327′680 vertices)
**Max image voxel resolution as input**	0.5 x 0.5 x 0.5 mm^3^	1.0 x 1.0 x 1.0 mm^3^
**Average thickness**	1 mm thicker than Freesurfer in all the diagnostic groups and in all time points (baseline and month 24)	1 mm thinner than Civet in all the diagnostic groups and in all time points (baseline and month 24)
**Strengths**	*CROSS-SECTIONAL*	*CROSS SECTIONAL*
	1) Constant thinning progression in different disease stages	1) Thinning progression peaks earlier than Civet
	2) Weak to medium trend of correlation to both MMSE score and hippocampal volume	2) Slightly higher disease effect (Hedge’s g) in comparing CTR with pMCI and AD
	3) Sensitive in expected cortical regions affected by disease neuropathology (i.e.: cingulate, dorsolateral frontal and parietal cortex)	3) Higher, but not significant, AUC to discriminate CTR versus pMCI or AD
		4) Sensitive in expected but also scattered unexpected cortical regions affected by disease neuropathology
	*LONGITUDINAL*	*LONGITUDINAL*
	1) Higher disease effect in pMCI and AD	1) Higher disease effect trend in CTR
	2) More sensitive to significant atrophic patterns in frontal-parietal regions (especially in pMCI)	2) Better correlation with hippocampal volumetric atrophy
	3) Sensitive to detect statistical significant atrophic differences between: AD vs CTR; AD vs sMCI; pMCI vs CTR	3) Sensitive to detect statistical significant atrophic differences between: AD vs CTR; AD vs sMCI
	4) Sensitive enough to detect statistical significant atrophic differences in many temporal ROIs between: sMCI vs pMCI	4) Higher, but not significant, AUC to discriminate pMCI due to AD in a time span of 2 years
**Algorithm Validations**	Manual method of tagging GM/CSF and GM/WM interfaces of forty brains on twenty regions of interest of young healthy volunteers (Kabani et al. 2001)	Comparison against post-mortem subjects with Huntington Disease and healthy control (Rosas et al. 2002) both of 43 years old

Comparative table where the main characteristics of the pipelines involved in this head-to-head comparison are summarized. MINC: Medical Imaging Network Common Data Form; DICOM: Digital Imaging and Communications in Medicine; NIFTI: Neuroimaging Informatics Technology Initiative; MNI OBJ: geometry file format developed by the Montreal Neurological Institute; PIAL: geometry file format developed by Martinos Center for Biomedical Imaging.

### Study design

The workflow of the study is reported as supplementary figure (see S1 Fig.).

### MRI acquisition

The Alzheimer’s Disease Neuroimaging Initiative (ADNI) has a specific protocol for the acquisition and harmonization of MR images. The ADNI 3D T1-weighted structural images are acquired using selected systems from GE Healthcare, Philips Medical Systems and Siemens Medical Solutions, with an eye toward minimizing cross-platform differences. The Magnetization Prepared RApid Gradient Echo (MPRAGE) acquisition sequence has nominal T1 = 1000 ms, TR = 2400 ms and TE = 5 ms. The B2B acquisition set in ADNI1 is composed of a MPRAGE scan and a MPRAGE-repeat scan.

### Visual quality control

All the post-processed scans output by neuGRID and VIP were quality controlled by an expert evaluator, who visually inspected them using the Matlab Imaging toolbox for 3D surfaces, which enables the user to rotate, zoom in and out the cortical surface along all the possible orientations. A reconstructed mesh was judged accurate when all the following 23 Sulci were visible and correctly reconstructed: (I) Sylvian Fissure, (II) Central Sulcus, (III) Postcentral Sulcus, (IV) Precentral Sulcus, (V) Superior Temporal Sulcus, (VII) Intraparietal Sulcus, (VIII) Primary Intermediate Sulcus, (IX) Secondary Intermediate Sulcus, (X) Transverse Occipital Sulcus, (XI) Inferior Temporal Sulcus, (XII) Inferior Frontal Sulcus, (XII) Middle Frontal Sulcus, (XIV) Olfactory Sulcus, (XV) Occipital-Temporal Sulcus, (XVI) Collateral Sulcus, (XVII) Olfactory Control Line, (XVIII) Olfactory-Middle Frontal Control Line, (XIX) Middle Frontal-Precentral Control Line, (XX) Precentral-Central Control Line, (XXI) Central-Postcentral Control Line, (XXII) Postcentral-Transverse Occipital Control Line and (XXIII) Occipital Control Line. As a result of this visual QC, only one of the two B2B cortical surfaces was chosen for analyses.

### Hybrid Template Generation enabling head-to-head (H2H) comparison

**Fig 1:**
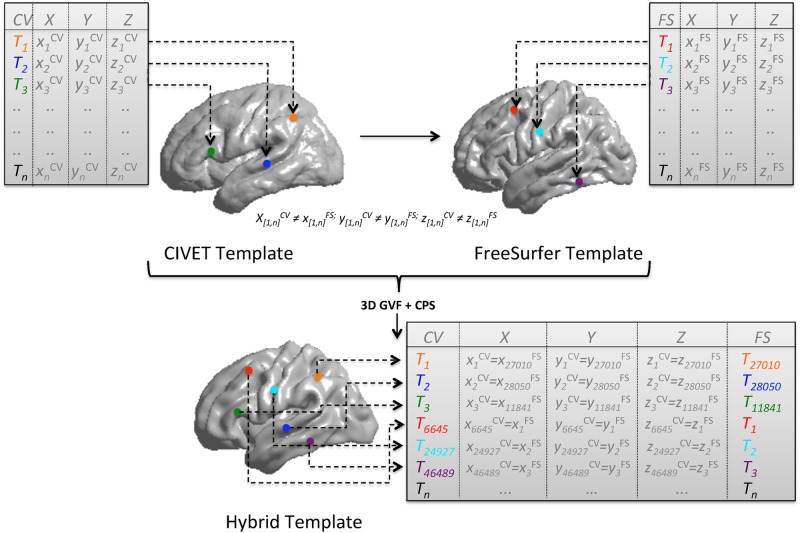
Registration of templates and surface points correspondence. Source template is Civet’s surface while target template is the Freesurfer’ surface template. Starting from two averaged surfaces (previously created from the same set of 10 CTR, 10 sMCI, and 10 AD brains) the hybrid template (characterized by 81924 vertices and 163840 faces) is derived after 15 GFV iterations. In GVF, deformations are achieved by tuning an underlying set of control points (187×187×187) in the source surface. Control point displacements are then interpolated to obtain a continuous transformation through basis spline functions. To keep the contour smooth, a membrane and percentage thin plate energy was used as regularization. The parameters defining the attraction to edges and energy surfaces were empirically determined. Finally, the CPS step defined the mutual correspondence of Civet and Freesurfer thickness values for each vertex. CV: Civet; FS: Freesurfer; X-Y-Z: value of the vertex space coordinates; T: value of the cortical thickness for each vertex; n: number of vertices (min = 0; max = 81924); 3D GVF: 3D gradient vector flow; CPS: Closest point search.

Cortex surfaces as extracted by Civet and Freesurfer are morphologically and topographically different. For an accurate comparison to be possible, it was necessary to deform the surface morphology of at least one algorithm. To map each point of one surface onto the other, we adopted an elastic non-rigid registration to get the right displacement vector. To our knowledge, Gradient Vector Flow (GVF) has not been used before to control 3D free form deformation. The vector field computed via GVF provided the directions along which each vertex of our source surface could evolve to match a corresponding point on the target surface. Once registered, space coordinates of each face vertices are coincident and vertices are spatially aligned. Subsequently, in order to compare the correct cortical index value at each vertex, we adopted the Closest Point Search (CPS) technique, essential to establish the correct topographical match of the same morphological points obtained with 3D GVF. For each point, CPS returned the mutual match between Civet’s and Freesurfer’s cortical thickness array. The entire process enabling the head-to-head comparison is illustrated in Fig. 1. The procedure was implemented using Matlab (v2009b). The data generated in this study are made publicly available to promote the evaluation of cortical thickness tool (https://neugrid4you.eu/datasets).

### Atlases and ROIs Definition

The head-to-head comparison and the ROI analyses between pipelines were done using the Harvard-Oxford cortical structural atlas. We chose 28 out of the 48 cortical areas provided [17], consistently with those used by other reference work groups [18–21]. For a complete list of the selected ROIs, see Table 3.

**Table 3:** Cross sectional ROI-based analysis.

BASELINE																
ROI			CIVET							FREESURFER						
			CTR VS SMCI		CTR VS pMCI		CTR VS AD		ANOVA	CTR vs SMCI		CTR VS pMCI		CTR VS AD		ANOVA
			Δ MEAN (mm) ± σ						P-value	Δ MEAN (mm) ± σ						P-value
**3**	**Frontal**	*Superior Frontal Gyrus*	*-0.10*	*0.36*	*-0.18*	*0.38*	*-0.17*	*0.34*	*N.S.*	*-0.09*	*0.32*	*-0.21*	*0.35*	*-0.19*	*0.33*	*N.S.*
**4**		*Middle Frontal Gyrus*	*-0.12*	*0.27*	*-0.18*	*0.29*	*-0.20*	*0.24*	*N.S.*	*-0.08*	*0.65*	*-0.20*	*0.68*	*-0.19*	*0.57*	*N.S.*
**5**		*Inferior Frontal Gyrus, pars triangularis*	*-0.10*	*0.41*	*-0.13*	*0.35*	*-0.13*	*0.34*	*N.S.*	*-0.06*	*0.36*	*-0.13*	*0.37*	*-0.13*	*0.37*	*N.S.*
**6**		*Inferior Frontal Gyrus, pars opercularis*	*-0.08*	*0.32*	*-0.12*	*0.26*	*-0.14*	*0.28*	*N.S.*	*-0.05*	*0.22*	*-0.15*	*0.23*	*-0.16*	*0.23*	*N.S.*
**33**		*Frontal Orbital Cortex*	*-0.10*	*0.41*	*-0.13*	*0.39*	*-0.22*	*0.37*	*N.S.*	*-0.08*	*0.38*	*-0.13*	*0.35*	*-0.15*	*0.38*	*N.S.*
**18**	**Parietal**	*Superior Parietal Lobule*	*-0.09*	*0.47*	*-0.16*	*0.48*	*-0.14*	*0.45*	*N.S.*	*-0.06*	*0.23*	*-0.21^π^*	*0.20*	*-0.17^Ω^*	*0.22*	*0.050*
**19**		*Supramarginal Gyrus, anterior division*	*-0.07*	*0.30*	*-0.14*	*0.29*	*-0.19*	*0.28*	*N.S.*	*-0.05*	*0.32*	*-0.18*	*0.31*	*-0.16*	*0.30*	*N.S.*
**20**		*Supramarginal Gyrus, posterior division*	*-0.10*	*0.27*	*-0.17*	*0.26*	*-0.23*	*0.27*	*N.S.*	*-0.08*	*0.33*	*-0.22*	*0.29*	*-0.21*	*0.33*	*N.S.*
**31**		*Precuneus Cortex*	*-0.08*	*0.43*	*-0.18*	*0.37*	*-0.18*	*0.38*	*N.S.*	*-0.10*	*0.41*	*-0.25*	*0.40*	*-0.20*	*0.37*	*N.S.*
**22**	**Ocp.**	*Lateral Occipital Cortex, superior division*	*-0.09*	*0.36*	*-0.18*	*0.35*	*-0.18*	*0.41*	*N.S.*	*-0.06*	*0.35*	*-0.23*	*0.34*	*-0.21*	*0.40*	*N.S.*
**23**		*Lateral Occipital Cortex, inferior division*	*-0.07*	*0.29*	*-0.15*	*0.32*	*-0.19*	*0.25*	*N.S.*	*-0.05*	*0.30*	*-0.17*	*0.30*	*-0.20*	*0.27*	*N.S.*
**29**	**Lmb.**	*Cingulate Gyrus, anterior division*	*-0.05*	*0.47*	*-0.11*	*0.44*	*-0.11*	*0.45*	*N.S.*	*-0.06*	*0.48*	*-0.08*	*0.50*	*-0.08*	*0.53*	*N.S.*
**30**		*Cingulate Gyrus, posterior division*	*-0.08*	*0.54*	*-0.21*	*0.55*	*-0.22*	*0.51*	*N.S.*	*-0.10*	*0.65*	*-0.23*	*0.66*	*-0.21*	*0.62*	*N.S.*
**8**	**Temporal**	*Temporal Pole*	*-0.17*	*0.55*	*-0.18*	*0.55*	*-0.38*	*0.67*	*N.S.*	*-0.14*	*0.73*	*-0.24*	*0.69*	*-0.33*	*0.71*	*N.S.*
**9**		*Superior Temporal Gyrus, anterior division*	*-0.10*	*0.50*	*-0.10*	*0.51*	*-0.23*	*0.59*	*N.S.*	*-0.07*	*0.99*	*-0.15*	*0.83*	*-0.20*	*0.90*	*N.S.*
**10**		*Superior Temporal Gyrus, posterior division*	*-0.14*	*0.45*	*-0.15*	*0.38*	*-0.24*	*0.44*	*N.S.*	*-0.12*	*0.62*	*-0.19*	*0.63*	*-0.23*	*0.61*	*N.S.*
**11**		*Middle Temporal Gyrus, anterior division*	*-0.13*	*0.51*	*-0.16*	*0.51*	*-0.31*	*0.48*	*N.S.*	*-0.12*	*0.74*	*-0.16*	*0.70*	*-0.25*	*0.70*	*N.S.*
**12**		*Middle Temporal Gyrus, posterior division*	*-0.13*	*0.29*	*-0.18*	*0.23*	*-0.33*	*0.25*	*N.S.*	*-0.11*	*0.61*	*-0.20*	*0.49*	*-0.28*	*0.58*	*N.S.*
**13**		*Middle Temporal Gyrus, temporo occipital part*	*-0.11*	*0.32*	*-0.20*	*0.35*	*-0.27*	*0.33*	*N.S.*	*-0.08*	*0.54*	*-0.23*	*0.54*	*-0.26*	*0.48*	*N.S.*
**14**		*Inferior Temporal Gyrus, anterior division*	*-0.11*	*0.59*	*-0.14*	*0.62*	*-0.30*	*0.56*	*N.S.*	*-0.11*	*0.81*	*-0.17*	*0.87*	*-0.25*	*0.74*	*N.S.*
**15**		*Inferior Temporal Gyrus, posterior division*	*-0.09*	*0.57*	*-0.15*	*0.59*	*-0.29*	*0.66*	*N.S.*	*-0.11*	*0.41*	*-0.21*	*0.41*	*-0.29*	*0.39*	*N.S.*
**16**		*Inferior Temporal Gyrus, temporo occipital part*	*-0.08*	*0.64*	*-0.13*	*0.57*	*-0.21*	*0.58*	*N.S.*	*-0.09*	*0.62*	*-0.18*	*0.63*	*-0.24*	*0.63*	*N.S.*
**34**		*Parahippocampal Gyrus, anterior division*	*-0.19*	*0.67*	*-0.28*	*0.67*	*-0.55*	*0.63*	*N.S.*	*-0.21*	*1.21*	*-0.33*	*1.22*	*-0.59*	*1.16*	*N.S.*
**35**		*Parahippocampal Gyrus, posterior division*	*-0.11*	*0.57*	*-0.15*	*0.59*	*-0.32*	*0.56*	*N.S.*	*-0.10*	*1.25*	*-0.15*	*1.22*	*-0.25*	*1.23*	*N.S.*
**37**		*Temporal Fusiform Cortex, anterior division*	*-0.14*	*0.57*	*-0.17*	*0.59*	*-0.35*	*0.52*	*N.S.*	*-0.15*	*0.80*	*-0.24*	*0.77*	*-0.41*	*0.73*	*N.S.*
**38**		*Temporal Fusiform Cortex, posterior division*	*-0.11*	*0.37*	*-0.11*	*0.38*	*-0.30*	*0.35*	*N.S.*	*-0.13*	*0.46*	*-0.21*	*0.44*	*-0.34*	*0.44*	*N.S.*
**45**		*HeschFs Gyrus (includes HI and H2)*	*-0.09*	*0.18*	*-0.12*	*0.18*	*-0.22*	*0.18*	*N.S.*	*-0.11*	*0.24*	*-0.15*	*0.26*	*-0.17*	*0.25*	*N.S.*
**46**		*Temporal Planum*	*-0.11*	*0.16*	*-0.13*	*0.14*	*-0.21^Ω^*	*0.14*	*0.041*	*-0.11*	*0.37*	*-0.17*	*0.38*	*-0.20*	*0.37*	*N.S.*

Cross-sectional average cortical thinning differences (mm), standard deviation (σ), and Tukey-Kramer multiple comparison post-hoc analysis in ANOVA (P). The data refer to three groups: (a) CTR versus sMCI, (b) CTR versus pMCI and (c) CTR versus AD; α = 0.05 level. Ω: Significant difference between “CTR versus sMCI” and “CTR versus AD”; Π: Significant difference between “CTR versus sMCI” and “CTR versus pMCI”. N.S.: Not significant; CTR: Normal elderly controls; sMCI: stable MCI; pMCI: progressive MCI; AD: Alzheimer’s disease.

### Statistical analysis to compare Cortical Thinning patterns

Cortical thinning within the same diagnostic groups was assessed using paired samples t-tests. P-maps were corrected for multiple comparisons using the False Discovery Rate (FDR; α = 0.01) method [22]. Tukey-Kramer post-hoc testing of ANOVA (α = 0.05 in cross sectional comparison and α = 0.01 in longitudinal analysis) was used to test thinning differences among the diagnostic groups and the different ROIs analyzed. Effect sizes were computed as Hedge’s g and Z-tests were performed to assess significant discrepancies between the performances of each pipeline. Correlations of cortical thickness to MMSE scores and hippocampal volumes were investigated, Steiger’s Z was used to assess significant differences between Pearson’s r values. Logistic regressions were applied on pre-selected thickness ROIs, and Receiver Operating Characteristic (ROC) curves were used to assess discriminative accuracy of the two pipelines. AUCs were statistically compared using the method adopted by Hanley and McNeil [23], setting the threshold for significance at a *p* value of 0.05. Kendall’s tau coefficients were calculated and the derived z-test converted into the Pearson’s correlation coefficient. Statistical analysis was performed with Matlab (v2009b).

### Cortical Metrics

Both pipelines define thickness as the Euclidean distance and both can produce maps not restricted to the original MRI voxel resolution: thus, they can detect sub-millimeter differences between and within groups [8,24]. For the sake of this article, we defined the concept of “disease effect” as the relative predominance of one pipeline over the other to detect atrophy when comparing two groups (G) or two time-points (T):

$$DISEASEEFFECT\left\{∆G|∆T\right\}=\left({∆}^{FS}Thickness-{∆}^{CV}Thickness\right)$$ (1)

**Fig 2:**
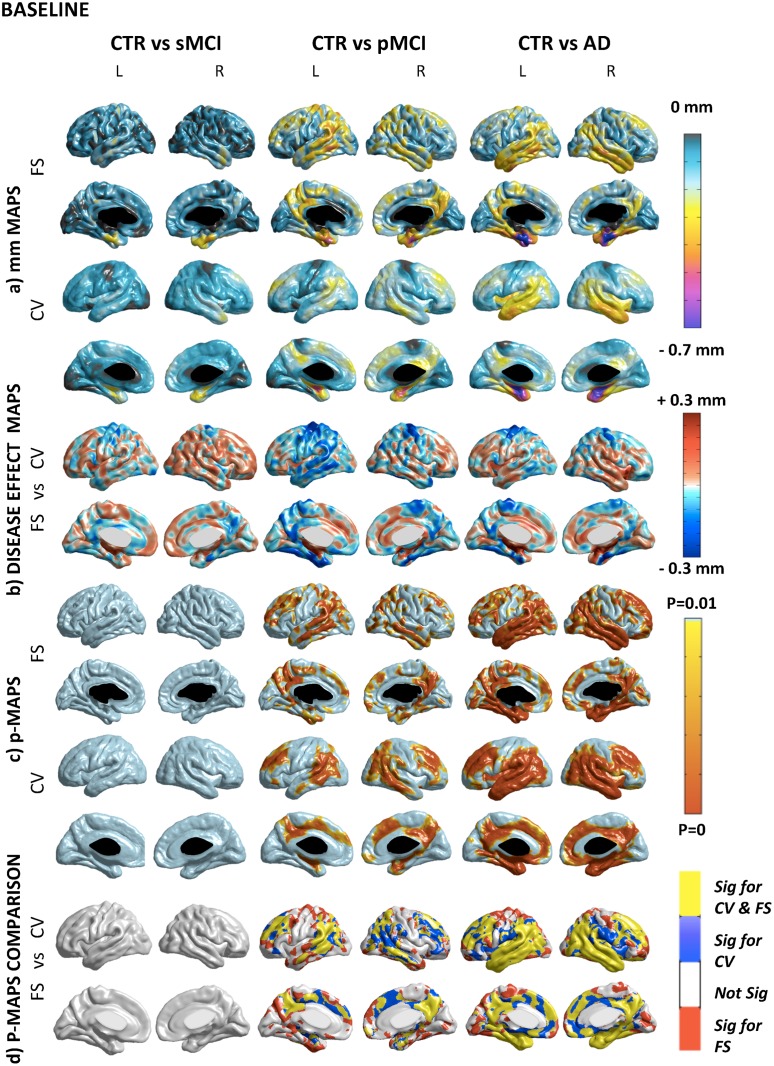
Cross-sectional comparison. A) Absolute difference maps (mm) in Freesurfer and Civet. The degree of atrophy ranges between 0.1 and 0.7 mm in the different areas of the cortical mantle. B) Disease effect maps. There is a consistent delta (±0.3 mm) among the compared groups. Negative value means higher disease effect for Freesurfer (i.e.: parietal-temporal and precuneus areas); positive value means higher disease effect for Civet (i.e.: association areas and limbic parts of the cortex). C) Statistical difference maps (p<0.01 FDR-corrected). No significant voxels were found comparing CTR to sMCI. Atrophic areas were found contrasting pMCI with CTR (i.e.: the posterior cingulate, temporal lobe and frontal gyrus) with both tools. Comparing CTR versus AD the statistical significance extended (i.e.: medial temporal, retrosplenial, and lateral temporal regions). D) Overlapping and not-overlapping atrophic regions are shown. Significant voxels detected by both pipelines are in yellow; voxels detected only by Civet are in blue; voxels detected only by Freesurfer are in red. CV: Civet; FS: Freesurfer; L: Left hemisphere; R: Right hemisphere; CTR: Normal elderly controls; sMCI: stable MCI; pMCI: progressive MCI; AD: Alzheimer’s Disease.

**Fig 3:**
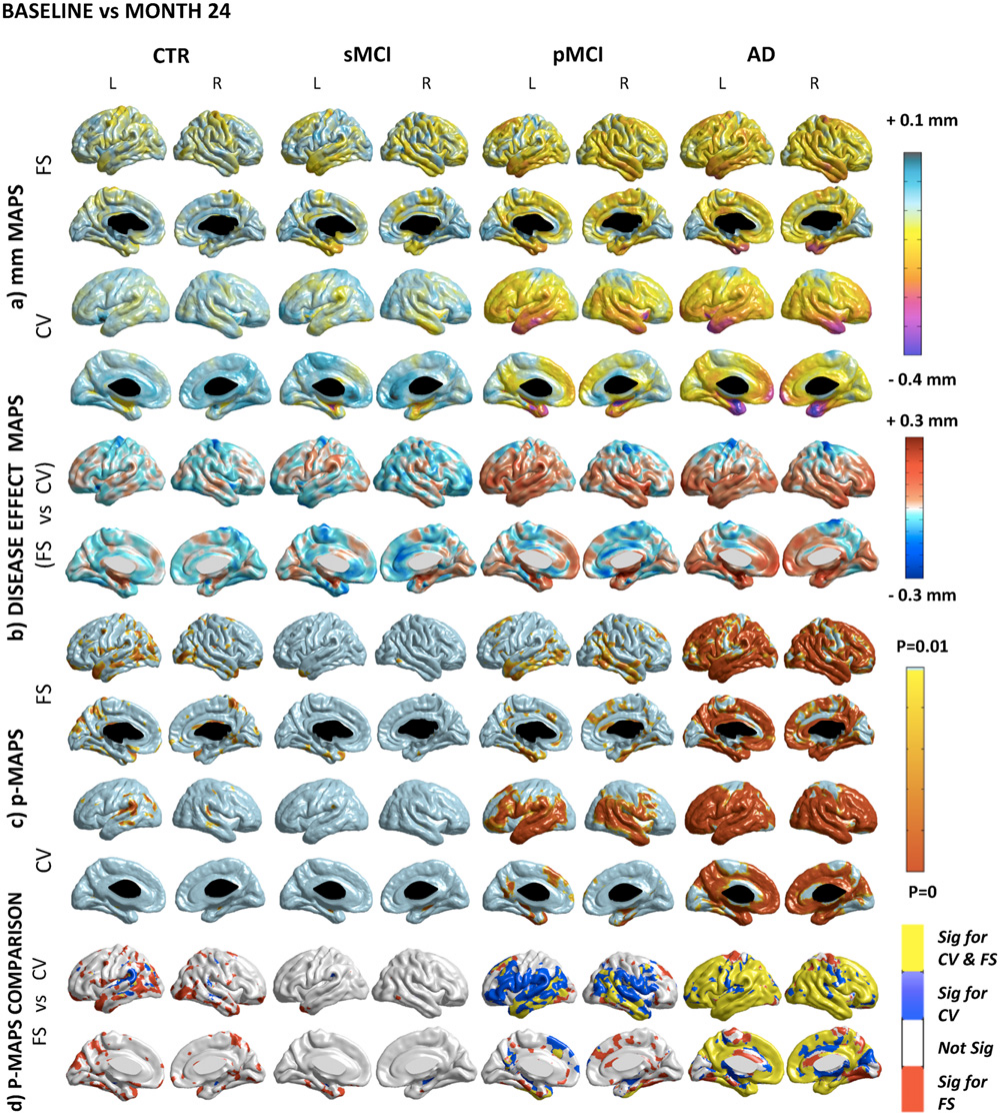
Longitudinal comparison. A) Absolute difference maps (mm) in each group. In CTR and sMCI, both pipelines report a very mild and widespread cortical thinning rate in the motor, somatosensory, verbal and visual association cortex. In pMCI, the atrophy peaks at rates around 0.3 mm in the medial temporal cortex, temporal-parietal-frontal neocortices, with sparing of the sensorimotor strip and of the visual cortex. In AD, the atrophy in the same areas accelerates beyond 0.4 mm. B) Disease effect maps. The mean estimate of the longitudinal disease effect in CTR and sMCI as computed by Freesurfer is greater, although Civet shows higher results in few scattered areas. Furthermore, in the entire disease spectrum, Freesurfer exhibited higher disease effect in the motor cortex. In pMCI, Civet exhibits a greater disease effect except for the cingulate gyrus, while in the AD group the exception is represented by the precuneus. C) Statistical difference maps (p<0.01 FDR-corrected). In CTR, Civet detects an atrophic cluster in the angular gyrus; while Freesurfer in the precuneus and in the temporo-occipital lobe. The pattern in sMCI was more reduced than in CTR. In pMCI Freesurfer was not able to find many regions detected by Civet with the same significance and extension (i.e.: orbital, triangulal, and opercular portion of the inferior frontal gyrus, transverse-temporal and mesial part of the superior frontal cortex, inferior parietal cortex, the superior temporal gyrus). Freesurfer was more sensitive in few scattered expected and unexpected regions. For both pipelines, the longitudinal AD shrinkage showed significant areas throughout the temporal, frontal and parietal lobes, consistently with the progression of the disease. Some shrivelling differences were detected in the anterior division of the cingulate, in the limbic lobe and in the cuneus. D) Overlapping and not-overlapping atrophic regions are shown. Significant voxels detected by both pipelines are in yellow; voxels detected only by Civet are in blue; voxels detected only by Freesurfer are in red. CV: Civet; FS: Freesurfer; L: Left hemisphere; R: Right hemisphere; CTR: Normal elderly controls; sMCI: stable MCI; pMCI: progressive MCI; AD: Alzheimer’s Disease.

The values of the disease effect are mapped vertex by vertex on the hybrid template previously created (see Figs. 2 and 3 panel b).

## Results

### Comparison of cortical metrics

The reconstruction of cortical thickness from B2B scans provided identical outcomes within the same pipeline (see S2 Fig.).

Compared to Civet, Freesurfer provided absolute values systematically lower by about 30% (see S3 Fig.). The difference between Civet and Freesurfer with respect to between-subjects variability (CoV) [25] ranges between 17–26% in the different diagnostic groups. The whole cortical thickness value at baseline and at month 24 is reported as S2 Table; both Civet and Freesurfer showed increasing values of thinning rates with the progression of the pathology. The relative percentage of thinning in paired diagnostic groups at baseline is reported as S3 Table; no statistical differences among the groups were detected in neither pipelines. The percentage of longitudinal thinning rate across the four different diagnostic groups is reported as S4 Table; both pipelines detected differences between AD versus CTR, and between AD versus sMCI; moreover, Civet was able to detect a significant longitudinal thinning difference between pMCI versus CTR.

### Cross-sectional and longitudinal thinning differences between Civet and Freesurfer

Fig. 2 compares CTR with sMCI, pMCI, and AD at baseline, and shows the details of the differences between Civet and Freesurfer at the individual vertex level. Fig. 3 compares, for each diagnostic group, the longitudinal (2 years) cortical thinning rate at the individual vertex level as computed by Civet and Freesurfer.

### ROI Analysis

Table 3 represents the comparison of the cross-sectional thickness differences at baseline, while Table 4 represents the longitudinal thinning rates with respect to the 28 selected ROIs. Cross-sectionally, the multiple comparison procedure highlighted small differences. Civet indicated as significant the temporal planum ROI, while Freesurfer identified as significant the superior parietal lobe. Longitudinally, Civet appeared to be much more sensitive in detecting significant thinning rate differences between CTR and AD in all the 28 ROIs considered, as opposed to only 22 ROIs as detected by Freesurfer (check symbol ¥). Comparing sMCI to AD, Civet was able to detect significant longitudinal thinning rate changes in all the 28 ROIs, compared to only 7 ROIs in Freesurfer (check symbol ◉). Again, Civet was able to detect significant longitudinal thinning rate changes between CTR and pMCI in 18 ROIs, as opposed to only 10 ROIs in Freesurfer (check symbol ¢). Lastly, Civet detected significant longitudinal thinning rate changes also between sMCI and pMCI in 10 ROIs (check symbol Ξ) while Freesurfer could not find any variations. P values for multiple comparisons were always more significant in Civet (P < 0.0001).

**Table 4:** Longitudinal ROI-based analysis.

			BASELINE VS MONTH24																	
ROI			CIVET									FREESURFER								
			CTR		sMCI		pMCI		AD		ANOVA P-value	CTR		sMCI		pMCI		AD		ANOVA P-value
			△ MEAN (mm) ± σ									△ MEAN (mm) ± σ								
3	***Frontal***	*Superior Frontal Gyrus*	*0.00*	*0.08*	*−0.04*	*0.12*	*−0.10*	*0.12*	*−0.13* ^*¥*^	^*⊙*^ *0.12*	*<0.0001*	*−0.04*	*0.10*	*−0.06*	*0.13*	*−0.12*	*0.14*	*−0.13* ^*¥*^	*0.12*	*0.0002*
4		*Middle Frontal Gyrus*	*−0.03*	*0.06*	*−0.05*	*0.10*	*−0.11* ^*¢*^	*0.12*	*−0.13* ^*¥*^	^*⊙*^ *0.12*	*<0.0001*	*−0.04*	*0.08*	*−0.06*	*0.13*	*−0.09*	*0.12*	*−0.11* ^*¥*^	*0.13*	*0.0031*
5		*Inferior Frontal Gyrus, pars triangularis*	*−0.03*	*0.08*	*−0.03*	*0.11*	*−0.12* ^*¢*^	^*Ξ*^ *0.11*	*−0.15* ^*¥*^	^*⊙*^ *0.13*	*<0.0001*	*−0.04*	*0.08*	*−0.05*	*0.15*	*−0.07*	*0.10*	*−0.09*	*0.11*	*N.S.*
6		*Inferior Frontal Gyrus, pars opercularis*	*−0.02*	*0.07*	*−0.04*	*0.10*	*−0.11* ^*¢*^	*0.10*	*−0.14* ^*¥*^	^*⊙*^ *0.12*	*<0.0001*	*−0.05*	*0.11*	*−0.05*	*0.15*	*−0.07*	*0.12*	*−0.11*	*0.11*	*N.S.*
33		*Frontal Orbital Cortex*	*−0.12*	*0.10*	*−0.03*	*0.15*	*−0.11* ^*¢*^	*0.14*	*−0.15* ^*¥*^	^*⊙*^ *0.16*	*<0.0001*	*−0.04*	*0.07*	*−0.05*	*0.11*	*−0.08*	*0.11*	*−0.10* ^*¥*^	*0.09*	*0.0011*
18	***Parietal***	*Superior Parietal Lobule*	*−0.03*	*0.09*	*−0.02*	*0.10*	*−0.06*	*0.11*	*−0.09* ^*¥*^	^*⊙*^ *0.10*	*0.0006*	*−0.05*	*0.10*	*−0.05*	*0.14*	*−0.06*	*0.11*	*−0.08*	*0.10*	*N.S.*
19		*Supramarginal Gyrus, anterior division*	*−0.03*	*0.65*	*−0.04*	*0.09*	*−0.10* ^*¢*^	*0.10*	*−0.12* ^*¥*^	^*⊙*^ *0.11*	*<0.0001*	*−0.05*	*0.11*	*−0.04*	*0.10*	*−0.09*	*0.13*	*−0.12* ^*¥*^	*0.12*	*0.0022*
20		*Supramarginal Gyrus, posterior division*	*−0.04*	*0.07*	*−0.05*	*0.10*	*−0.12* ^*¢*^	*0.10*	*−0.14* ^*¥*^	^*⊙*^ *0.11*	*<0.0001*	*−0.05*	*0.09*	*−0.05*	*0.12*	*−0.09*	*0.12*	*−0.12* ^*¥*^	^*⊙*^ *0.11*	*0.0010*
31		*Precuneus Cortex*	*−0.01*	*0.07*	*−0.02*	*0.07*	*−0.07*	*0.11*	*−0.11* ^*¥*^	^*⊙*^ *0.11*	*<0.0001*	*−0.05*	*0.08*	*−0.03*	*0.07*	*−0.09*	*0.10*	*−0.11* ^*¥*^	^*⊙*^ *0.10*	*<0.0001*
22	***Ocp.***	*Lateral Occipital Cortex, superior division*	*−0.03*	*0.07*	*−0.02*	*0.09*	*−0.09*	^*Ξ*^ *0.09*	*−0.11* ^*¥*^	^*⊙*^ *0.11*	*<0.0001*	*−0.04*	*0.10*	*−0.06*	*0.13*	*−0.12*	*0.14*	*−0.13* ^*¥*^	*0.12*	*0.0002*
23		*Lateral Occipital Coitex, inferior division*	*−0.03*	*0.09*	*−0.03*	*0.11*	*−0.12* ^*¢*^	*0.11*	*−0.15* ^*¥*^	^*⊙*^ *0.15*	*<0.0001*	*−0.06*	*0.07*	*−0.05*	*0.10*	*−0.10*	*0.10*	*−0.07*	*0.09*	*N.S.*
29	***Lmb.***	*Cingulate Gyrus, anterior division*	*0.00*	*0.08*	*0.01*	*0.13*	*−0.04*	*0.12*	*−0.11* ^*¥*^	^*⊙*^ *0.14*	*<0.0001*	*−0.02*	*0.07*	*−0.04*	*0.11*	*−0.07*	*0.08*	*−0.08* ^*¥*^	*0.09*	*0.0024*
30		*Cingulate Gyrus, posterior division*	*−0.01*	*0.10*	*−0.02*	*0.07*	*−0.07*	*0.16*	*−0.12* ^*¥*^	^*⊙*^ *0.12*	*<0.0001*	*−0.04*	*0.06*	*−0.04*	*0.07*	*−0.10* ^*¢*^	*0.11*	*−0.11* ^*¥*^	^*⊙*^ *0.09*	*<0.0001*
8	***Temporal***	*Temporal Pole*	*−0.04*	*0.11*	*−0.05*	*0.19*	*−0.20* ^*¢*^	*0.17*	*−0.27* ^*¥*^	^*⊙*^ *0.29*	*<0.0001*	*−0.06*	*0.07*	*−0.09*	*0.11*	*−0.13*	*0.15*	*−0.18* ^*¥*^	*0.14*	*<0.0001*
9		*Superior Temporal Gyrus, anterior division*	*−0.03*	*0.07*	*−0.04*	*0.11*	*−0.16* ^*¢*^	^*Ξ*^ *0.10*	*−0.16* ^*¥*^	^*⊙*^ *0.11*	*<0.0001*	*−0.04*	*0.08*	*−0.06*	*0.10*	*−0.09*	*0.11*	*−0.12* ^*¥*^	*0.10*	*0.0002*
10		*Superior Temporal Gyrus, posterior division*	*−0.04*	*0.08*	*−0.05*	*0.11*	*−0.16* ^*¢*^	^*Ξ*^ *0.11*	*−0.16* ^*¥*^	^*⊙*^ *0.11*	*<0.0001*	*−0.04*	*0.08*	*−0.06*	*0.14*	*−0.08*	*0.10*	*−0.10*	*0.11*	*N.S.*
11		*Middle Temporal Gyrus, anterior division*	*−0.03*	*0.08*	*−0.06*	*0.13*	*−0.18* ^*¢*^	^*Ξ*^ *0.13*	*−0.21* ^*¥*^	^*⊙*^ *0.15*	*<0.0001*	*−0.04*	*0.07*	*−0.07*	*0.14*	*−0.12* ^*¢*^	*0.12*	*−0.10* ^*¥*^	*0.10*	*<0.0001*
12		*Middle Temporal Gyrus, posterior division*	*−0.04*	*0.08*	*−0.06*	*0.12*	*−0.19* ^*¢*^	^*Ξ*^ *0.15*	*−0.20* ^*¥*^	^*⊙*^ *0.12*	*<0.0001*	*−0.04*	*0.07*	*−0.06*	*0.12*	*−0.13* ^*¢*^	*0.12*	*−0.14* ^*¥*^	^*⊙*^ *0.11*	*<0.0001*
13		*Middle Temporal Gyrus, temporo occipital part*	*−0.04*	*0.06*	*−0.04*	*0.10*	*−0.16* ^*¢*^	^*Ξ*^ *0.12*	*−0.16* ^*¥*^	^*⊙*^ *0.11*	*<0.0001*	*−0.05*	*0.07*	*−0.06*	*0.12*	*−0.12*	*0.13*	*−0.11* ^*¥*^	*0.10*	*0.0015*
14		*Inferior Temporal Gyrus, anterior division*	*−0.02*	*0.08*	*−0.07*	*0.13*	*−0.18* ^*¢*^	*0.13*	*−0.21* ^*¥*^	^*⊙*^ *0.20*	*<0.0001*	*−0.04*	*0.06*	*−0.08*	*0.12*	*−0.13* ^*¢*^	*0.12*	*−0.14* ^*¥*^	*0.11*	*<0.0001*
15		*Inferior Temporal Gyrus, posterior division*	*−0.03*	*0.09*	*−0.07*	*0.13*	*−0.18* ^*¢*^	^*Ξ*^ *0.15*	*−0.19* ^*¥*^	^*⊙*^ *0.18*	*<0.0001*	*−0.05*	*0.07*	*−0.08*	*0.10*	*−0.12* ^*¢*^	*0.12*	*−0.14* ^*¥*^	^*⊙*^ *0.11*	*<0.0001*
16		*Inferior Temporal Gyrus, temporo occipital part*	*−0.02*	*0.09*	*−0.03*	*0.10*	*−0.15* ^*¢*^	^*Ξ*^ *0.14*	*−0.16* ^*¥*^	^*⊙*^ *0.15*	*<0.0001*	*−0.05*	*0.08*	*−0.06*	*0.09*	*−0.11*	*0.12*	*−0.12* ^*¥*^	*0.09*	*<0.0001*
34		*Parahippocampal Gyrus, anterior division*	*−0.05*	*0.13*	*−0.12*	*0.15*	*−0.18*	*0.16*	*−0.27* ^*¥*^	^*⊙*^ *0.27*	*<0.0001*	*−0.04*	*0.06*	*−0.08*	*0.08*	*−0.11* ^*¢*^	*0.10*	*−0.14* ^*¥*^	^*⊙*^ *0.10*	*<0.0001*
35		*Parahippocampal Gyrus, posterior division*	*−0.03*	*0.16*	*−0.07*	*0.09*	*−0.11*	*0.14*	*−0.17* ^*¥*^	^*⊙*^ *0.21*	*0.0002*	*−0.02*	*0.05*	*−0.04*	*0.07*	*−0.08* ^*¢*^	*0.08*	*−0.07* ^*¥*^	*0.08*	*0.0001*
37		*Temporal Fusiform Cortex, anterior division*	*−0.02*	*0.15*	*−0.06*	*0.15*	*−0.15*	*0.12*	*−0.24* ^*¥*^	^*⊙*^ *0.30*	*<0.0001*	*−0.04*	*0.08*	*−0.07*	*0.09*	*−0.14* ^*¢*^	*0.09*	*−0.17* ^*¥*^	^*⊙*^ *0.13*	*<0.0001*
38		*Temporal Fusiform Cortex, posterior division*	*−0.01*	*0.17*	*−0.03*	*0.11*	*−0.14*	*0.12*	*−0.20* ^*¥*^	^*⊙*^ *0.26*	*<0.0001*	*−0.05*	*0.08*	*−0.07*	*0.12*	*−0.15* ^*¢*^	*0.11*	*−0.17* ^*¥*^	*0.12*	*<0.0001*
45		*Heschl’s Gyrus (includes H1 and H2)*	*−0.05*	*0.07*	*−0.06*	*0.11*	*−0.13* ^*¢*^	*0.10*	*−0.15* ^*¥*^	^*⊙*^ *0.12*	*<0.0001*	*−0.05*	*0.11*	*−0.06*	*0.16*	*−0.09*	*0.11*	*−0.08*	*0.12*	*N.S.*
46		*Temporal Planum*	*−0.05*	*0.08*	*−0.05*	*0.11*	*−0.14* ^*¢*^	^*Ξ*^ *0.10*	*−0.15* ^*¥*^	^*⊙*^ *0.12*	*<0.0001*	*−0.05*	*0.09*	*−0.04*	*0.12*	*−0.08* ^*¢*^	*0.10*	*−0.08* ^*¥*^	*0.11*	*N.S.*

Longitudinal average cortical thinning differences (mm), standard deviation (σ), and Tukey-Kramer multiple comparison post-hoc analysis in ANOVA (P). The data refer to: (d) CTR, (e) sMCI, (f) pMCI and (g) AD; α = 0.01 level.

¢: Significant difference between CTR and pMCI;

¥: Significant difference between CTR and AD.

Ξ: Significant difference between sMCI and pMCI;

¤: Significant difference between sMCI and AD N.S.: Not significant; CTR: Normal elderly controls; sMCI: stable MCI; pMCI: progressive MCI; AD: Alzheimer's disease.

### Effect sizes

**Fig 4:**
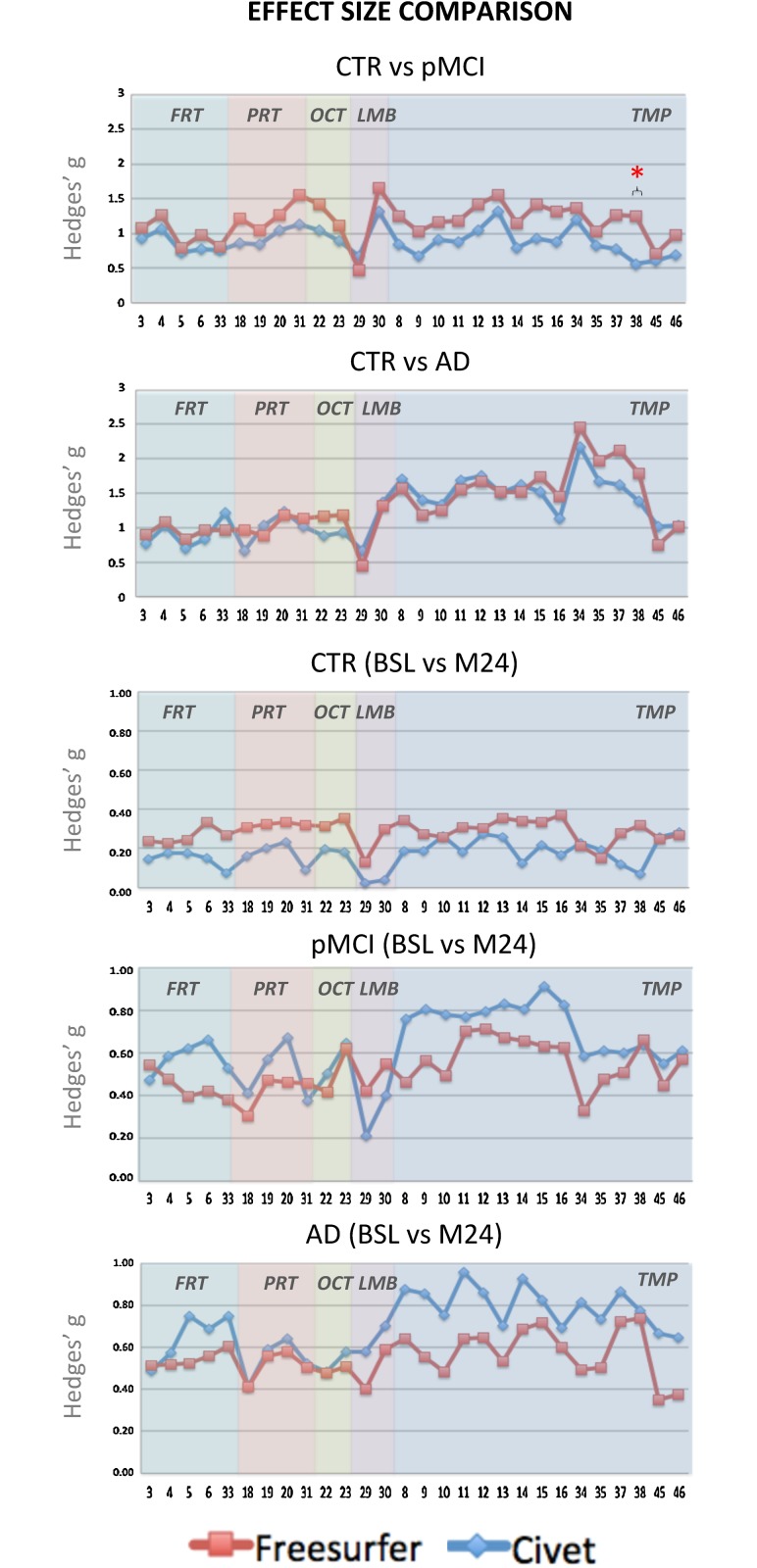
Hedges’ g effect size graphs in the different ROI areas. The first two panels represent the cross-sectional effect sizes comparing the overall trend of CTR versus pMCI, and of CTR versus AD. The remaining three panels represent the longitudinal effect sizes between the baseline and month 24 in CTR, pMCI, and AD groups. The * symbol stands for p<0.05.

The effect sizes were derived as the Hedge’s g (Fig. 4). In the cross-sectional analysis, we decided to represent only CTR versus pMCI and versus AD, being these the combinations of highest interest when defining populations for disease-modifying and clinical trials. The effect size was always above 0.8 in those cortical regions expected to be heavily affected by the disease neuropathology. In CTR versus pMCI, Freesurfer’s effect size was always higher. Only the posterior division of the temporal fusiform cortex was found to be statistically different (p<0.05) between the two pipelines. In CTR versus AD, the Hedge’s g values followed the same trend for both algorithms without any statistical difference.

Longitudinally, Hedge’s g trends were pretty similar for the two algorithms and increasing with the disease progression. No statistical differences were found in any ROIs or groups.

### Cortical thickness versus cognitive impairment and hippocampal volumetry

**Fig 5:**
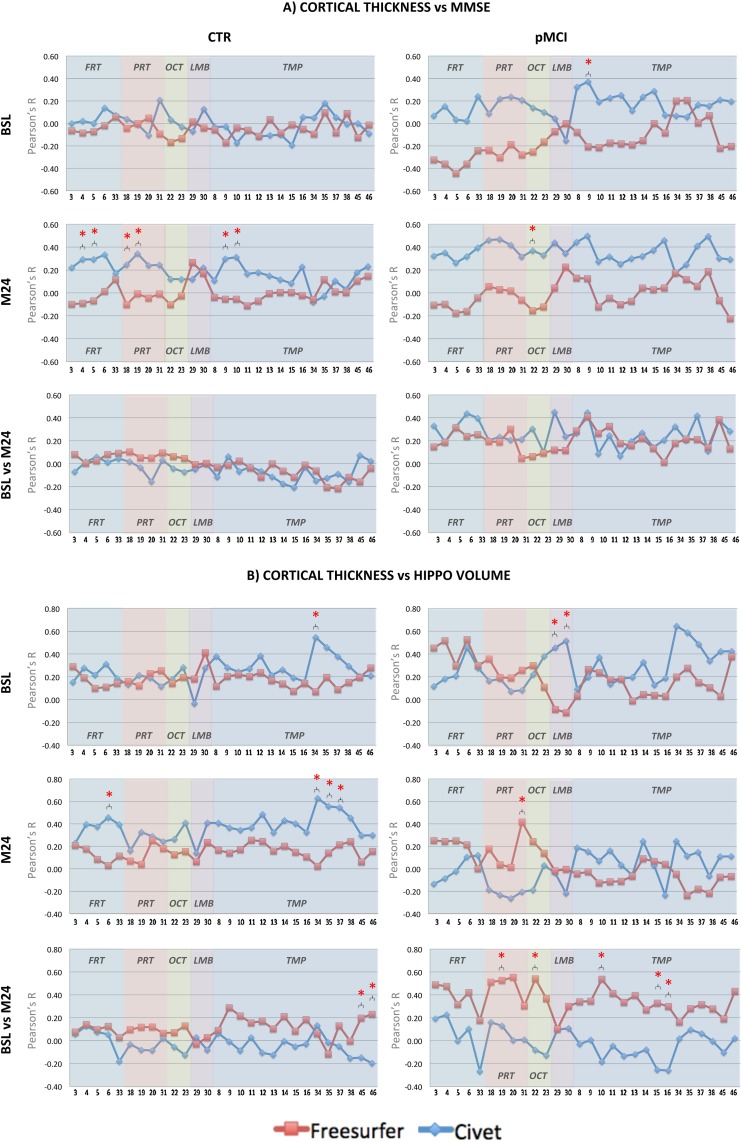
Pearson’s r coefficient of cortical thickness versus MMSE scores (panel A). In the CTR group, no significant differences between ROIs were detected in the two pipelines at BSL. At M24, significant differences between the two pipelines were found in the: middle frontal gyrus; inferior frontal gyrus—pars triangularis; superior parietal lobule; anterior division of the supramarginal gyrus; anterior and posterior division of the superior temporal gyrus. Longitudinally, no significant differences between ROIs were detected in the two pipelines. In the pMCI group, significant difference between the two pipelines was found at BSL in the: anterior division of the superior temporal gyrus. At M24, significant difference between the two pipelines was found in the: superior division of the lateral occipital cortex. Longitudinally, no significant differences between ROIs were detected in the two pipelines. **Pearson’s r coefficient of cortical thickness versus NeuroQuant hippocampal volume (panel B)**: In the CTR group, significant difference between the two pipelines at BSL was found in the: anterior division of the parahippocampal gyrus. At M24, significant differences between the two pipelines were found in the: inferior frontal gyrus—pars opercularis; anterior and posterior division of the parahippocampal gyrus; anterior division of the temporal fusiform cortex. Longitudinally, significant differences between the two pipelines were found in the: Heschl’s gyrus and temporal planum. In the pMCI group, significant difference between the two pipelines was found at BSL in the: precuneus cortex. Longitudinally, significant differences between the two pipelines were found in the: anterior division of the supramarginal gyrus, superior division of the lateral occipital cortex, posterior division of the superior temporal gyrus, posterior division of the inferior temporal gyrus, temporo-occipital part of the inferior temporal gyrus. In panels A and B, * symbol stands for p<0.05 (Steiger’s z-test). Red coloured lines represent the trends in Freesurfer, blue lines in Civet. CTR.: CTR: Normal elderly controls; sMCI: stable MCI; pMCI: progressive MCI; AD: Alzheimer’s disease; BSL: baseline; M24: month 24; FRT: Frontal; PRT: Parietal; OCT: Occipital; LIMB: Limbic; TMP: Temporal.

Pearson’s r correlation coefficients of regional cortical thickness with MMSE scores and quantitative hippocampal volume measurements (NeuroQuant—[26]) were investigated in each ROI (see Fig. 5 panels A and B) within the CTR and pMCI patients, which represent the most appropriate population for innovative clinical trial designs.

In the CTR group, the relationship between pipelines’ cortical thickness and cognitive function or hippocampal atrophy was generally weak (-0.2 < r < 0.2), cross-sectionally and longitudinally. This was expected due to the absence of the disease in these completely asymptomatic subjects. However, significant differences between Civet and Freesurfer were found in few areas (i.e.: frontal, parietal, occipital, and temporal).

In pMCI, the product momentums grew up to a medium and high levels (-0.27 < r < 0.64) especially for some expected ROIs, such as: precuneus cortex, cingulate and parahippocampal gyri. Significant differences between Civet and Freesurfer were found in a number of ROIs (i.e.: frontal, parietal, occipital, limbic, and temporal). Both Civet and Freesurfer cortical thickness measurements correlate better with hippocampal atrophy measurements than with neuropsychological tests.

### ROC Analysis

**Fig 6:**
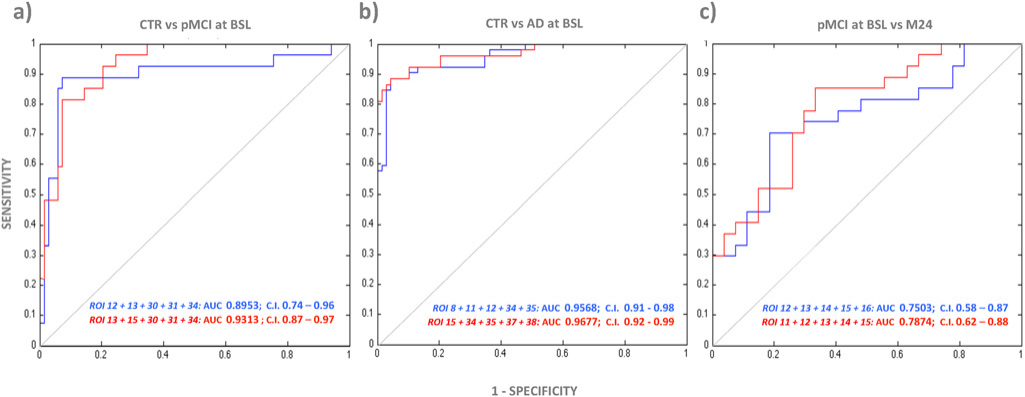
Receiving Operator Characteristic (ROC) curves showing the performances of Civet and Freesurfer in classifying: A) CTR versus pMCI at baseline; B) CTR versus AD at baseline; and C) pMCI at baseline from month 24. AUC with 95% CIs are reported for both Freesurfer in red and Civet in blue. CTR: Normal elderly controls; sMCI: stable MCI; pMCI: progressive MCI; AD: Alzheimer’s Disease; BSL: baseline; M24: month 24; AUC: Area Under the Curve; C.I: Confidence Interval; ROI 8: temporal pole; ROI 11: anterior division of the middle temporal gyrus; ROI 12: posterior division of the middle temporal gyrus; ROI 13: temporo-occipital part of middle temporal gyrus; ROI 15: posterior division of inferior temporal gyrus; ROI 16: temporo-occipital part of inferior temporal gyrus; ROI 30: posterior division of the cingulate gyrus; ROI 31: Precuneus Cortex; ROI 34: anterior division of the parahippocampal gyrus; ROI 35: posterior division of the parahippocampal gyrus; ROI 37: anterior division of the temporal fusiform cortex; ROI 38: posterior division of the temporal fusiform cortex.

Fig. 6 shows the Receiver Operating Characteristic (ROC) curves used to discriminate pMCI and AD patients from the CTR group at baseline, together with the longitudinal cortical pattern used to discriminate pMCI. Identifying the most informative ROI was mandatory to reduce the dimensionality problem. In order to maximize the discriminatory power, we adopted a sequential forward search strategy (i.e., adding successive ROIs to the target set) as feature selection criterion. The goal was to find the best combination of ROIs for both tools with the highest discriminatory power. The best ROIs used to generate the final ROCs were different in each curve and for each algorithm. We started selecting those ROI with the highest effect size; at each further step, we assessed other ROIs with a medium-large effect size (d > 0.8 in cross sectional analysis; d > 0.6 in longitudinal analysis). This process reduced the inherent noise of high-resolution data, as well as the risk of over-fitting. Logistic regressions on regional cortical thickness in the selected combinations of ROIs were performed to build ROC curves, AUCs and the relative Intervals of Confidence (CI). No statistical difference (p>0.05) was found between the AUCs derived with Civet and those derived with Freesurfer. At baseline, CTR versus pMCI yielded 0.8953 and 0.9313 (z = -0.46, r = 0.31), while CTR versus AD yielded 0.9568 and 0.9677 respectively (z = -0.38, r = 0.46). In the longitudinal framework, pMCI yielded 0.7503 and 0.7874 (z = -0.34, r = 0.21). Freesurfer performed slightly better in terms of classification accuracy, both on cross sectional and longitudinal analyses.

## Discussion

This study could be considered as a first attempt to verify the mutual strengths and weaknesses of Civet and Freesurfer in a real head-to-head challenge, at the precision level of the single voxel. In the literature, only phantom-based validation methods have been used [27,28] but this kind of approach does not take into consideration every aspects of real data. We investigated and compared the performances of Civet and Freesurfer when applied to the same ADNI1 groups which included subjects on the entire disease spectrum, as monitored in a 2-year time frame. The analyses showed commonalities and differences.

Civet and Freesurfer are characterized by specific and distinctive procedures, making it difficult to compare their outputs. This problem was solved adopting a combined approach, applying both the GVF and CPS to ensure a robust comparison of meshes characterized by different morphometry and topography completely different. Thanks to the direct vertex-by-vertex cross-algorithm comparison, the differences between the two algorithms, with regard both to cross-sectional and longitudinal analysis, were analytically mapped.

Differences between thickness evaluation of the first test (MPRAGE) and that of the retest (MPRAGE-Repeat) did not appear, suggesting high repeatability. Both Civet’s and Freesurfer’s performances changed according to the disease stage, pointing out that neither algorithm can be considered better than the other, or the best acting. Freesurfer systematically underestimated the absolute thickness by about 1 mm if compared to Civet’s performance. Explanations for this evidence are not trivial. However, the restriction of Freesurfer to 1.0 mm as resolution for the volumes to be processed could be one possible reason. Civet, relying on the volumetric Laplacian approach, can use higher resolutions (e.g.: 0.8 or 0.9 mm) often adopted in ADNI1. An important role might be also played by the different mathematical procedures used by the two tools when reconstructing the gray matter sheet. Moreover, the skeleton reconstruction method adopted by Civet to build the GM sheet tends to overestimate the cortical thickness in case of blurred regions (i.e.: regions affected by noise where CSF volume is small); on the other hand, Freesurfer relies on the inner white deformation surface approach, which can be strongly influenced by the anatomical accuracy of the surface reconstruction at both inner and outer boundaries, thus giving a partially unfair anatomical accuracy of the surface reconstruction and assessment of the cortical thickness.

Cross-sectionally, both algorithms were sensitive to cortical thinning in those cortical regions heavily affected by the neuropathology. Comparing CTR to pMCI, the regions of significance found by both tool were overlapping with the those found comparing CTR and AD, albeit smaller, indicating that the differences in cortical thinning are progressive and well detectable even before a formal diagnosis of AD. This means that both tools can detect the characteristic signature of AD. Both Civet and Freesurfer were able to efficiently differentiate CTR from the AD and pMCI. All the ROIs granting such a good discrimination rate belonged to the temporal lobe. An interesting consideration for future works is the possibility to use Civet and Freesurfer to differentiate AD in particular subclasses, namely familial AD, early onset AD, and late onset AD [29,30].

Longitudinally, both pipelines showed more statistically atrophic clusters in CTR than in sMCI, but this should be considered as a confounding phenotypic effect due to demographic, numerosity, clinical and other genetic characteristics. Further analyses with a larger sample will be conducted to clarify this particular behaviour. In pMCI, Civet was able to highlight a characteristic atrophic pattern involving expected temporal areas, such as the inferior margin of central gyrus and extended lateral frontal-parietal areas, as expected. The Civet’s higher effect size and its more representative cortical signature suggest that this tool can detect the typical atrophic patterns in subject that will convert to AD within 2 years more efficiently. In the discriminant analysis, Civet produced an AUC slightly lower than that produced by Freesurfer; but this was probably due to random noises that confuses classifiers, producing changes hard to predict and control. Additional explanation can be related to the fact that longitudinally, on a vertex-by-vertex basis, Civet showed a more extensive effect than Freesurfer, while on a ROI basis the differences between the pipelines were not significant. In the AD cohort both Freesurfer and Civet were analogously sensitive to the thinning patterns. As far as the correlation between the cortical thinning and hippocampal atrophic rate is concerned, Freesurfer showed a better trend, probably due to the exploitation of the longitudinal stream.

Given its progressive alteration along the MCI-to-AD course, cortical thickness seems to be a promising neuroimaging candidate marker. With few exceptions, the two algorithms showed robust multi-ROI correlation patterns fairly consistent with the usual clinical and regional neuroimaging biomarkers, thus producing new, 3D, global profiles of the disease progression.

Ultimately, having reliable 3D diagnostic markers would enable clinicians to identify and treat MCI patients who will evolve into AD patients in a timely manner, as disease-modifying treatments will become available.

Future studies, including the MR 3.0 Tesla field strength, additional time points, extended age range of subject, larger and additional groups, might be helpful to further address the spatial and temporal atrophic pattern of the Alzheimer’s changes.

Freesurfer and Civet have been validated against either histological analysis or manual measurements [31–34], but none of them has been contrasted against different stages of the Alzheimer’s pathology. Future works should focus on further validating both pipelines against a database of cortical thickness derived from a population of normal and abnormal cadaveric brains, such as those recently defined in the BigBrain initiative (https://bigbrain.loris.ca/).

Some limitations should be considered in the interpretation of the present results. First, the tools here described need to be further compared with other recent available techniques, such as: Toads-Cruise [35], ARCTIC [36], MILXCTE [37], DiReCT [38], or CLADA [39]. Second, as expert manual rater in neuroimaging represents the gold standard, independent evaluators should compare the performance and accuracy of each automatic pipeline. Third, each tool should be validated against harmonized MR datasets, such as: standardized ADNI analysis dataset [40], WW-ADNI [41], AddNeuroMed [42] and OASIS [43]. Fourth, computational time is worth consideration: the extensive use of Civet or Freesurfer to analyse large volumes of data mandatorily requires HPC, Grid or Cloud resources, due to the protracted processing time needed. Additional developing and programming can make these algorithms more reliable, faster and slighter.

## Conclusion

Both Civet and Freesurfer demonstrated high sensitivity to cortical gray matter changes cross-sectionally and longitudinally. Additional efforts are needed to clarify the ability of these tools to address particular clinical and research questions concerning the future use of cortical thickness as a biomarker, and in particular their ability to: (I) predict cortical decline along different time points, (II) reduce the number of patients needed for future clinical trials, (III) help monitoring the efficacy of disease modifying drugs.

## Supporting Information

S1 Fig Flowchart of the study methodology. (TIF)

S2 Fig Civet and Freesurfer B2B repeatability. (TIF)

S3 Fig Freesurfer and Civet absolute cortical thickness maps for every diagnostic class. (TIF)

S1 Table List of subjects’ RIDs. (XLSX)

S2 Table Whole brain absolute mean cortical thickness (mm) ± standard deviation (σ) for each diagnostic group at baseline and month 24. (TIF)

S3 Table Cross-sectional thinning percentages (%) ± standard deviation (σ) in paired diagnostic groups at baseline. (TIF)

S4 Table Longitudinal thinning percentage (%) ± standard deviation (σ) in each diagnostic group in a time span of two years. (TIF)
