# Supplementary figures and images for: Head-to-Head Comparison of Two Popular Cortical Thickness Extraction Algorithms: A Cross-Sectional and Longitudinal Study
Source: PLoS One. 2015 Mar 17;10(3):e0117692. doi: 10.1371/journal.pone.0117692 (PMC4364123; doi:10.1371/journal.pone.0117692)

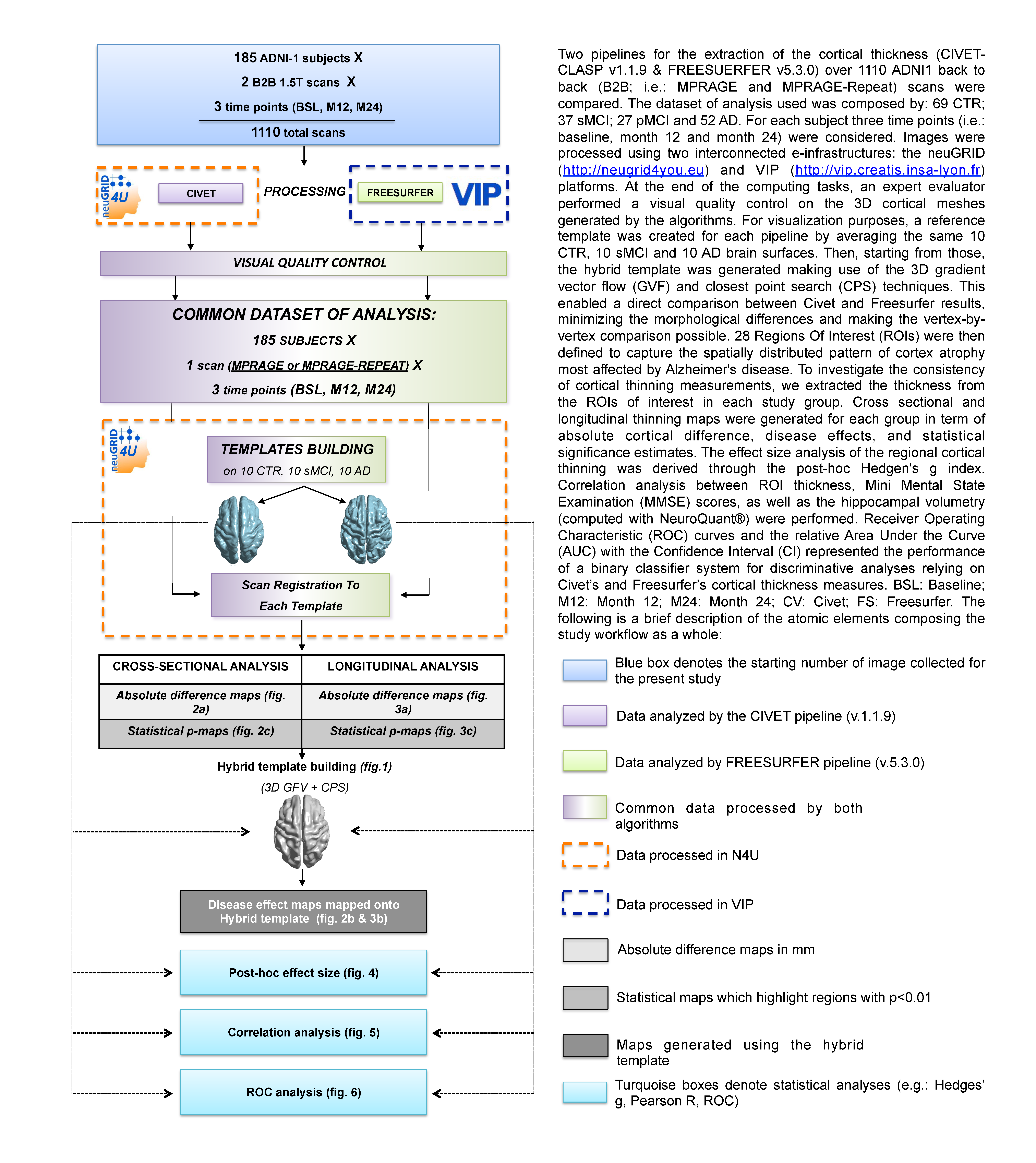

Supplement: S1 Fig — (TIF) [file pone.0117692.s001.tif]

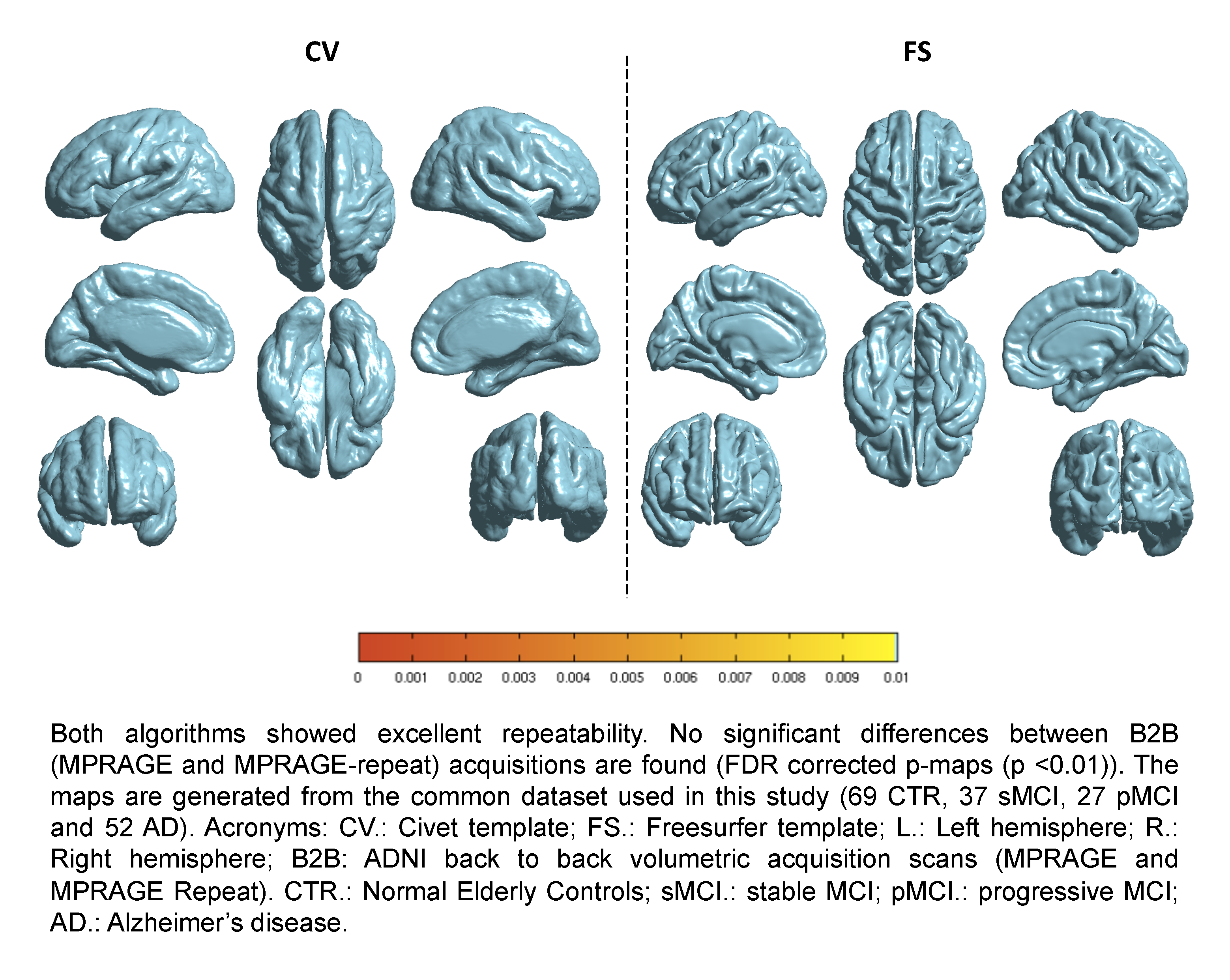

Supplement: S2 Fig — (TIF) [file pone.0117692.s002.tif]

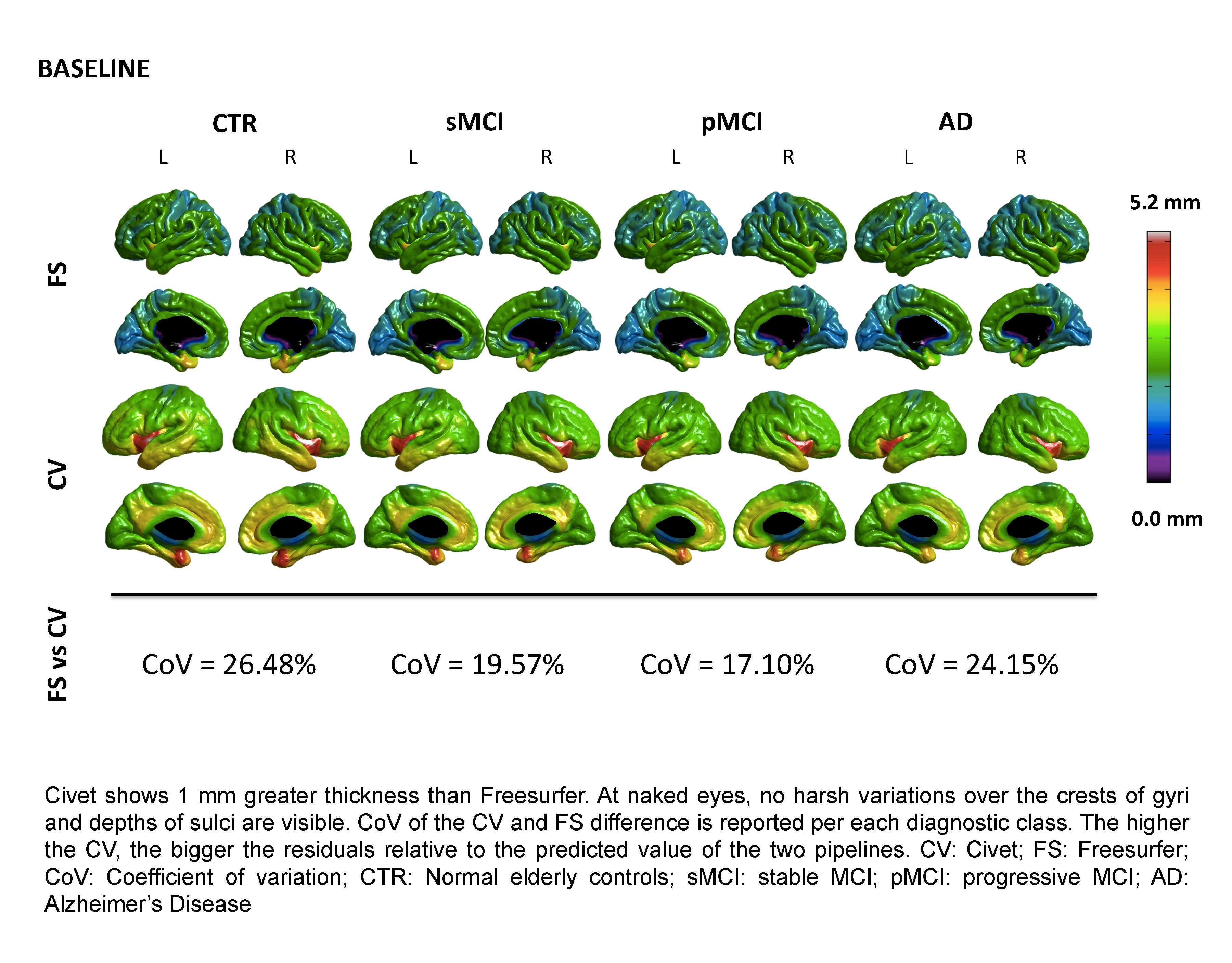

Supplement: S3 Fig — (TIF) [file pone.0117692.s003.tif]

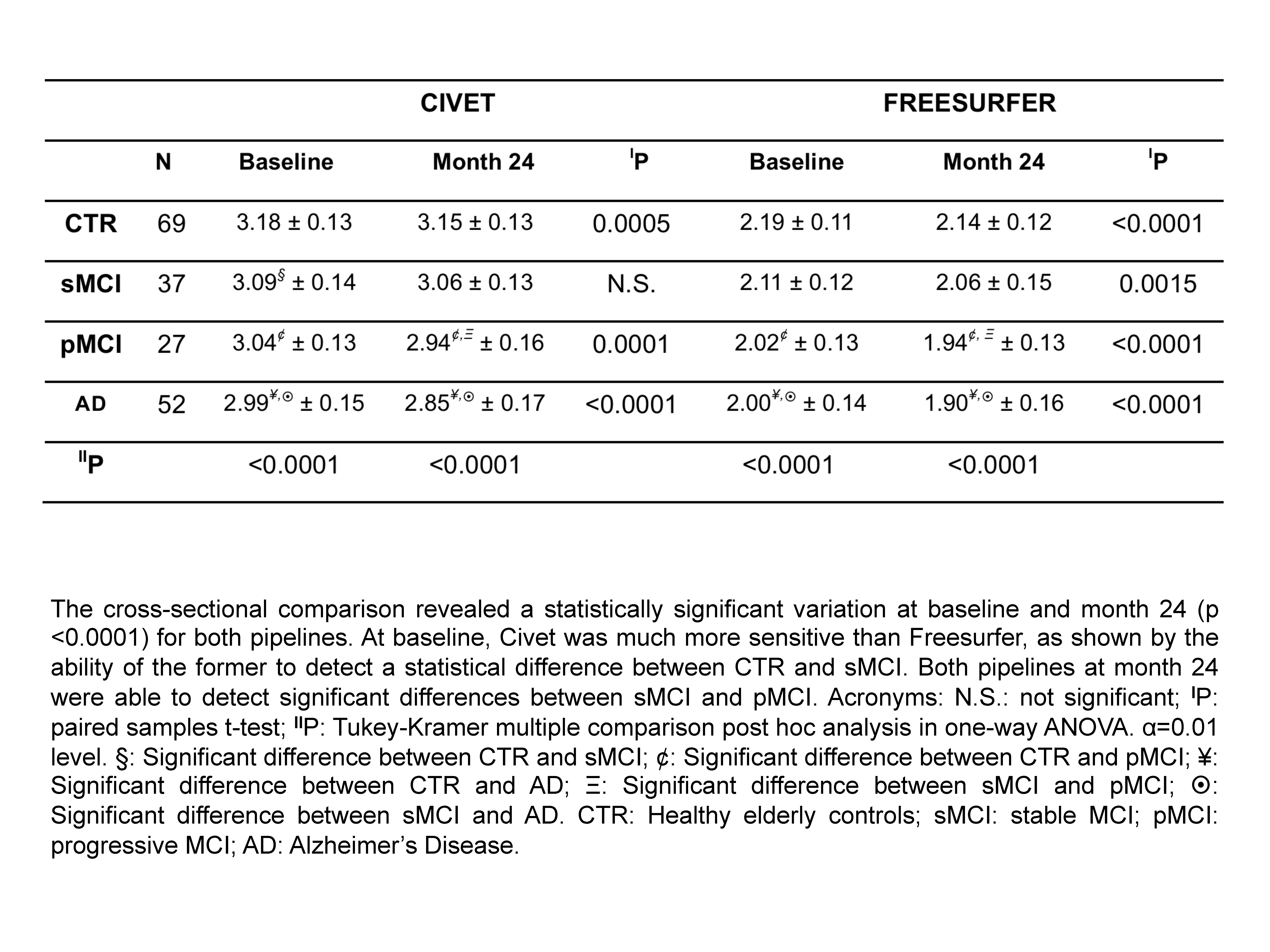

Supplement: S2 Table — (TIF) [file pone.0117692.s005.tif]

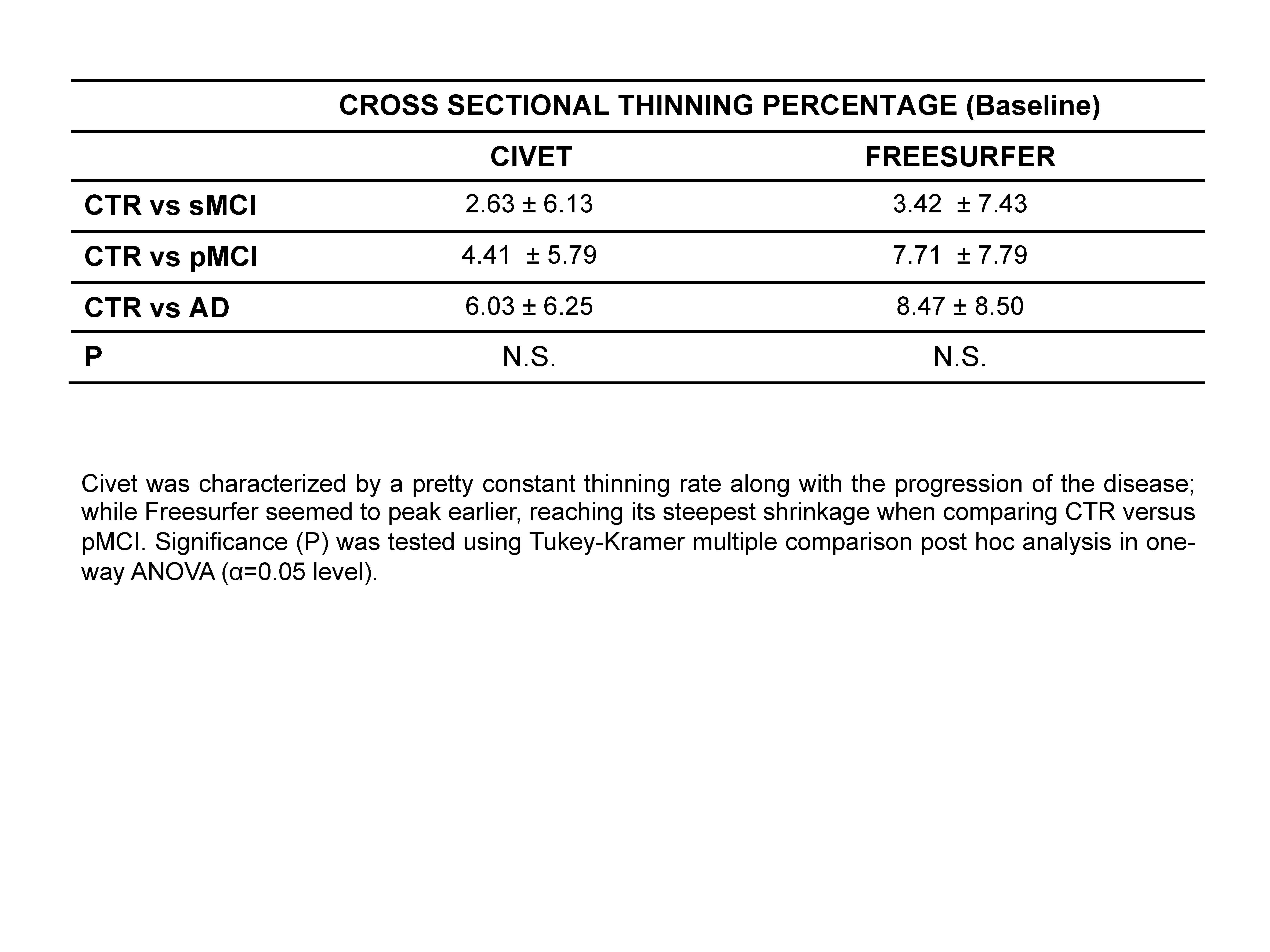

Supplement: S3 Table — (TIF) [file pone.0117692.s006.tif]

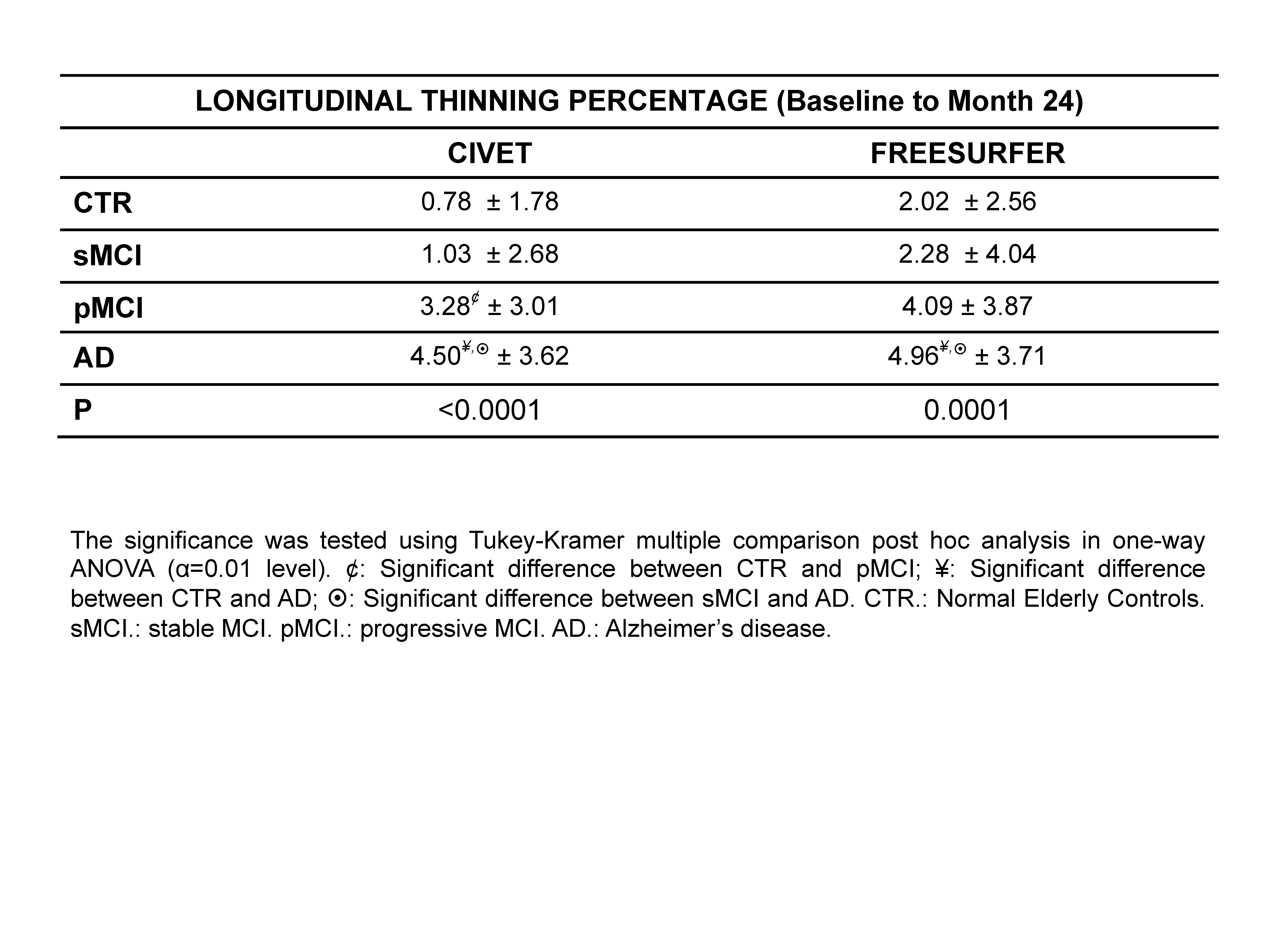

Supplement: S4 Table — (TIF) [file pone.0117692.s007.tif]
